# Post COVID-19 condition and its physical, mental and social implications: protocol of a 2-year longitudinal cohort study in the Belgian adult population

**DOI:** 10.1186/s13690-022-00906-2

**Published:** 2022-06-04

**Authors:** Pierre Smith, Kristiaan Proesmans, Dieter Van Cauteren, Stefaan Demarest, Sabine Drieskens, Robby De Pauw, Laura Cornelissen, Karin De Ridder, Rana Charafeddine

**Affiliations:** 1 Department of Epidemiology and Public Health, Sciensano, Brussels, Belgium; 2 Institute of Health and Society (IRSS), Université Catholique de Louvain, Brussels, Belgium; 3 Department of Rehabilitation Sciences, Ghent University, Ghent, Belgium

**Keywords:** COVID-19, Post COVID-19 condition, Health, Symptoms, Longitudinal study

## Abstract

**Background:**

Since the onset of the COVID-19 pandemic, most research has focused on the pathophysiology and management of the acute symptoms of COVID-19, yet some people tend to experience symptoms beyond the acute phase of infection, that is, Post COVID-19 condition (PCC). However, evidence on the long-term health impacts of a COVID-19 infection are still scarce. The purpose of this paper is to describe the COVIMPACT study, which aims to set up a cohort of people who have been tested positive for COVID-19 and study the evolution of their physical, mental and social health over the medium (3 months) and long term (two years), and the factors associated with an (un)favorable evolution.

**Methods:**

COVIMPACT is a longitudinal cohort study organised over a two-years period between April 2021 and April 2023. The eligible population is all people aged 18 years and older, living in Belgium, with a recent COVID-19 infection and contacted by the health authorities for contact tracing. Two questionnaires are used: a baseline questionnaire that aims to assess the initial health status of the participants and their status during the acute phase of the illness, and a follow-up questionnaire that is sent every three months after participants enter into the cohort. A matched non-COVID-19 control group was also selected. As of November 1, 2021, 10,708 people completed the baseline questionnaire (5% of the eligible population) and the follow-up participation rate was 79%. In total, 48% of the cohort participants appeared to fit the proposed case definition of PCC (i.e. report at least one symptom related to their COVID-19 infection three months afterwards).

**Discussion:**

This study was designed to provide timely information on the short and long term impact of a COVID-19 infection, to stakeholders such as policymakers, health practitioners and people with PCC. Although the follow-up participation rate was good (79%), the participation rate of the eligible population was low (5%). Compared to other studies, this study has a large sample, of non-hospitalised and hospitalised people, who will be followed over a long period of 3 months to two years post infection, and with a global approach to their health.

**Supplementary Information:**

The online version contains supplementary material available at 10.1186/s13690-022-00906-2.

## Background

In 2020, the world faced the emergence of a new pathogen, named the novel severe acute respiratory syndrome coronavirus 2 or SARS-CoV-2. On the 11th of March 2020, the World Health Organization (WHO) qualified COVID-19 as a global pandemic. Since the start of this pandemic, most of the research has focused on the pathophysiology and management of acute symptoms associated with COVID-19 infection [1, 2]. These acute symptoms can manifest in different systems including the respiratory, gastrointestinal, muscular and neurological systems with varying degrees of severity [3, 4]. However, some people continue to experience symptoms beyond the acute phase of infection. Consequently, long COVID has appeared as a new term in the literature to describe the long term symptoms of a COVID-19 infection [5].

The term long COVID comes from the COVID-19 patients calling themselves “long haulers” because they still experience symptoms months after recovering from the acute phase of the infection [5]. This phenomenon was recently defined by the WHO as Post COVID-19 condition (PCC) [6]. With the growing number of people infected with COVID-19, PCC is becoming an important public health issue. A systematic review found that the prevalence of PCC varies considerably between studies, from 5 to 80%, depending on the definition used in terms of duration, but also on the symptoms included [7]. Indeed, our understanding of PCC remains weak and there is currently no consensus on its definition, and this for three main reasons. First, PCC is relatively new and therefore little studied in the literature. Second, the study of PCC requires longitudinal data and a long-term follow-up of several months after COVID-19 infection, making data collection challenging. Third, people suffering from PCC report a plethora of quite heterogeneous symptoms [8, 9]. Due to the disparity in clinical symptoms, it is suggested [1, 10] that PCC is due to different clinical mechanisms, the three main hypotheses being: (1) medium and long-term consequences of SARS-CoV-2 organ damage, (2) post-intensive care syndrome, and (3) long-term COVID-19 syndrome. Regarding the first mechanism, organ damage following acute COVID-19 infection such as myocardial infection, stroke, acute encephalitis, neuromuscular disorders, or renal failure, may lead to persistent symptoms. Regarding the second mechanism, the stay in the intensive care unit can lead to impaired muscles and nerves, mental health problems and cognitive impairment, leading to persistent symptoms. Regarding the long-term COVID-19 syndrome, the main biological hypothesis is the hyper-inflammatory cytokine storm, a prolonged pro-inflammatory response related to SARS-CoV-2 infection inducing an atypical response of the immune system and mast cells, and a cascade of heterogeneous symptoms [10]. The National Institute for Health and Care Excellence (NICE) has suggested the following clinical case definition of PCC: *“ when signs and symptoms developed during or after an infection consistent with COVID-19, continue for more than 12 weeks and are not explained by an alternative diagnosis”* [11].

A recent meta-analysis [8] of 33 studies on COVID-19 survivors showed that 46% had at least one symptom three months after acute infection. Another meta-analysis found that the most common symptoms of PCC were fatigue (58%), headache (44%), attention disorder (27%), and dyspnea (24%) [12]. Finally, some studies have also shown that PCC is not evenly distributed among people infected with COVID-19, with a higher risk among women [13], people with pre-existing comorbidities [14], and people hospitalised following COVID-19 [15]. However, these meta-analyses also highlighted limitations in existing studies on PCC. First, most of the studies on PCC have been carried out on patients hospitalised during the acute phase of COVID-19 infection who were followed after their discharge from hospital [16–18]. Yet, PCC also affects people with moderate acute symptoms not requiring hospitalisation, and even people who were asymptomatic during the acute phase of the infection [14, 19, 20]. As the majority of COVID-19 patients will not require hospitalisations, it is essential to assess and understand the distribution, patterns, and risk factors of PCC among non-hospitalised patients. Second, PCC not only has an impact on the physical health of individuals, but also on other dimensions of health. Indeed, the symptoms of PCC can cause disabilities in daily life that will disrupt the social and professional life of individuals and have a negative impact on their mental, social and economic health [21]. However, longitudinal studies that have assessed the impact of PCC on these different dimensions are limited. Third, most studies had a follow-up between 30 and 90 days after infection or hospitalisation, so the course of persistent symptoms and health status beyond that remains unclear. A meta-analysis [22] identified the studies with a follow-up of one year, three studies were carried out on a sample of both hospitalised and non-hospitalised people [23–25] and two on a non-hospitalised population [26, 27]. However, these studies had a limited sample size, with the smallest being 83 people and the largest being 543 people. Fourth, there is a lack of case–control studies or studies with a matched non-COVID-19 group [1]. Some symptoms of PCC are common to other infections and health problems in the general population (e.g. fatigue and headaches), so a control group is essential to know if these symptoms but also their impact on the physical, mental and social health is solely due to the COVID-19 infection and PCC.

In this context, a longitudinal cohort study called COVIMPACT was set up in April 2021 by Sciensano, the Belgian institute of public health, to systematically contact the adults in Belgium who have been tested positive with for COVID-19. Participants are followed up in order to study the evolution of their physical, mental, and social health over the long term. Their health situation will be compared to a similar socio-demographic sample of the general population not infected with COVID-19. In this paper we describe the methods of the COVIMPACT study and discuss its strengths and limitations.

### Study objectives and hypothesis

The main objective of the COVIMPACT study is to assess, among people with a recent COVID-19 infection, the evolution of their physical, mental and social health over the medium (min 3 months) and long term (max two years), and the factors associated with an (un)favorable evolution.

The secondary objectives of the study are to:

- (1) Assess the type and duration of PCC symptoms over time (i.e. between 3 months and two years after infection) and in the different follow-up periods (i.e. according to different COVID-19 variants and seasons).
- (2) Assess the determinants of short (min 3 months) and long term (max 2 years) PCC symptoms such as underlying chronic conditions, severity of the infection, hospitalisation, socioeconomic status, vaccination status, or health behaviours.
- (3) Assess the short and long term evolution of (a) quality of life, (b) mental health, (c) functional limitations, and (d) social contacts and employment among people with PCC, compared to those without persistent COVID-19 symptom and to a similar socio-demographic sample of the general population not infected with COVID-19.

## Methods

### Setting

At the end of January 2020, the Belgian authorities reported the identification of a new coronavirus in Wuhan, China. In March 2020, the number of COVID-19 cases in Belgium increased exponentially. Between January 2020 and November 2021 Belgium faced four peaks of the COVID-19 pandemic: the first peak from March to April 2020, the second between September and December 2020, the third between March and April 2021 and the fourth peak started in October 2021 and was still increasing in November 2021. This study began at the end of April 2021, at the end of the third peak of the pandemic. Belgium started in January 2021 with the COVID-19 vaccination and by November 2021, 75% of the adult population was fully vaccinated. When the study was launched, the vaccination rate in the Belgian adult population was around 6%. At the start of the study the Alpha variant was dominant in Belgium (+—80%) and from July 2021 it was the Delta variant (+—99%) up to November 2021. Regarding the COVID-19 testing policy in Belgium, since May 2020, testing include all symptomatic individuals that fulfilled the case definition of a possible case (persons with respiratory symptoms), travellers and close contacts of positive cases [28, 29]. However, an online survey carried out in December 2021 by Sciensano, the Belgian Institute of Public Health, showed that only 35% of participants with mild to moderate symptoms of COVID-19 reported having taken a PCR test following their symptoms [30]. Finally, in November 2021, PCC was still not recognized as a disability in Belgium, but a working group has been formed to develop on a national care plan for people suffering from PCC.

### Study population and recruitment

The target population are people aged 18 years and older, living in Belgium (including non-citizens), with a recent COVID-19 infection confirmed via a laboratory test. In Belgium, when a COVID-19 test is positive, the laboratory sends the information to a central database “COVID-19 DATABASE” at Healthdata.be [31]. Based on this database, contact tracing call centers are automatically instructed to contact the COVID-19 cases and trace their contacts [29]. At the end of the call, the contact tracing agents inform the cases aged 18 and over about the study and ask them if they agree to receive more information about the study by SMS. If they agree, potential participants receive the SMS with a link to a website containing a description of the study and a link to the first baseline online questionnaire. The purpose of this procedure is to ensure the anonymity of participants, to avoid any perceived obligation to participate in the study and to be in compliance with data protection rules.

### Design

COVIMPACT is a longitudinal online cohort study organised over two years between April 2021 and April 2023. Potential participants will be recruited into the cohort until 3 months before the end of the study (January 2023). The study includes two types of online questionnaires: 1) a baseline questionnaire sent to participants at the time of their COVID-19 infection (i.e. after contact with the tracing center and 2) a follow-up questionnaire sent every three months following the person's entry into the cohort until the end of the study in April 2023. Therefore, depending on when the participants entered the study, the follow-up period can vary from 3 months to 2 years. The questionnaires are available in Dutch, French, German and English. The first baseline questionnaires were sent on April 29, 2021 and the first follow-up questionnaires on July 29, 2021. The link to the baseline questionnaire is sent to the potential participants by the contact tracing agents. Follow-up questionnaires are emailed to participants every 3 months by the research team with an automated system through LimeSurvey version 3.25 and R version 3.6.3 (2020–02-29) [32]. An automated R script was developed to daily extract the information provided by the participants on LimeSurvey, such as their email and the date of their participation in the baseline questionnaires. Afterwards, this information is used to generate a separate database in LimeSurvey with participants who have agreed to participate in the follow-up questionnaires. Individualised follow-up times were calculated for each of the participants based on the date of their participation in the baseline questionnaire. At each of these individualised follow-up times, participants are invited to participate in the follow-up questionnaire.

To encourage the participation of people in the cohort and reduce the loss to follow-up, infographics are produced every 3 months to present the main results of the study and shared in follow-up emails. Finally, two reminders are also automatically sent after one week and two weeks to people who have not completed the follow-up questionnaire or have only completed it partially. The follow-up system has been pre-tested and validated.

**Fig. 1:**
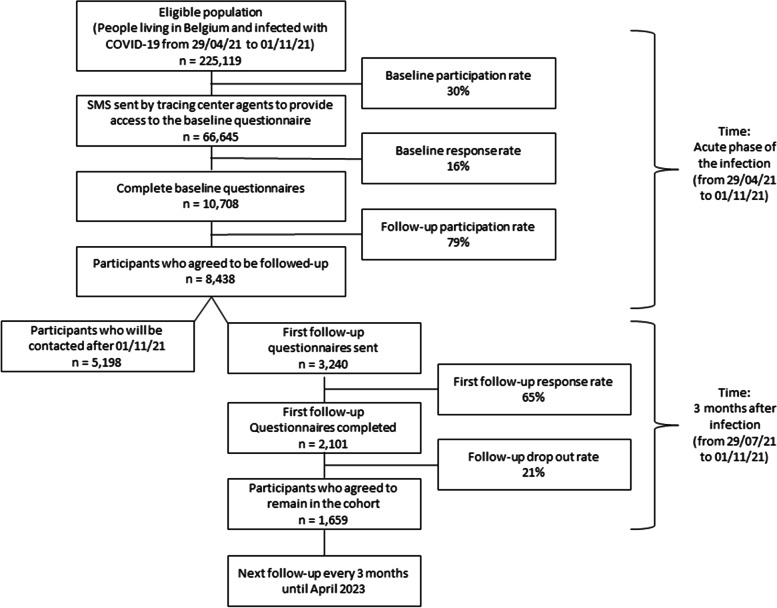
Flow diagram follow-up of participants between April 29 and November 1, 2021

The flow diagram of the study is presented in Fig. 1. The sample sizes and rates presented are calculated on the sample extracted on November 1, 2021, i.e. 6 months after the start of data collection (baseline and first 3-month follow-up questionnaires). Between April 29 and November 1 2021, 225,119 persons who tested positive for COVID-19 met the study's inclusion criteria. Among them, 66,645 (30%) agreed to receive an SMS with a link to a website containing the link to the baseline questionnaire. In total, 10,708 people completed the baseline questionnaire (16% of those who received the SMS, 5% of all eligible for inclusion). Among those who completed the baseline questionnaire, 8,438 (79%) agreed to be followed over time every 3 months and provided their email address. In total, 3,240 of the 8,438 people were contacted to complete the first 3-month follow-up questionnaire; the remaining 5,198 people will be contacted after November 1, 2021. The response rate to the first 3-month follow-up questionnaire was 65% (*n* = 2,101) and among the people who responded, 21% (*n* = 442) no longer wanted to participate in the follow-up.

### Consent

The consent of individuals to participate in the study was included and collected in the baseline questionnaire as well as in each follow-up questionnaire. In addition, at the end of each questionnaire participants could indicate whether they wished to be contacted or not for the next questionnaire. The study has been approved by the ethics committee of the Ghent university hospital (Commissie voor Medische Ethiek), B.U.N.: B6702021000287.

### Questionnaires and outcomes

Two questionnaires were submitted to the participants: a baseline questionnaire and follow-up questionnaires. Table 1 presents a summary of the variables collected and scales used in the baseline and follow-up questionnaires.

**Table 1:** Summary of the variables collected in the baseline and follow-up questionnaires

Modules	variables and scales	Baseline	Follow-up	Matched-control group (2018 BHIS)
**Sociodemographic information**	Demographics and socio-economic questions	X	X	X
	Change in employment and income status following the COVID-19 infection		X	
**COVID-19 variables**	Date of positive COVID-19 test	X		
	List of potential acute COVID-19 symptoms (yes / no)	X	X	
	Perceived recovery from COVID-19 infection from 1 (not at all) to 5 (completely)		X	
	List of potential medical complications of COVID-19 infection (yes / no)		X	
	List of potential post COVID-19 symptoms (yes / no)		X	
**Shortness of breath**	Medical Research Council (MRC) dyspnea scale	X	X	
**Fatigue**	Visual Analogue Scale for Fatigue from 0 (no fatigue) to 10 (very severe fatigue)	X	X	
**Vaccination**	COVID-19 vaccination (yes / no)	X	X	
	Number of doses and date of last dose	X	X	
	List of the different vaccines offered in Belgium (last vaccine received)	X	X	
**Health-related quality of life**	Modified EQ-5D (EuroQol Group)	X	X	X
**Functional limitations**	Washington city group (WCG)	X	X	X
	Global Activity Limitation Indicator (GALI)		X	X
**Mental health and wellbeing**	General Anxiety Disorder-7 (GAD-7)	X	X	X
	Patient Health Questionnaire-9 (PHQ-9)	X	X	X
	Visual Analogue Scale for Life Satisfaction from 0 (not at all satisfied) to 10 (totally satisfied)	X	X	X
**Social health**	UCLA Three-Item Loneliness Scale	X	X	X
**Health history and behaviours**	List of potential chronic diseases (yes / no)	X		X
	Mental health problem before COVID-19 infection (yes / no)	X		
	Tobacco smoking (frequency, quantity, number of years)	X		X
	Physical activity (frequency, duration)	X		X
	Food (consumption of fruits and vegetables, frequency)	X		X
	Body Mass Index	X		X
	Pregnancy (yes / no, if yes trimester)	X		X
	Since the COVID-19 infection change in the consumption of fruit and vegetables, alcohol, tobacco, sedatives, antidepressants (intensity)		X	
	Since the COVID-19 infection change in physical activity habits (intensity)		X	
**Health care use**	Hospitalisations since COVID-19 infection (type, number, duration)		X	
	Consultations with healthcare professionals since COVID-19 infection (type, number)		X	
	Diagnosis of post COVID-19 condition by a healthcare professional (yes / no)		X	

The baseline questionnaire aimed to evaluate (1) the health status of the participants before the infection (retrospectively) and (2) their status during the acute phase of the illness. This was done via the following topics: the symptoms experienced during the acute COVID-19 episode (if any), health-related quality of life (today and before COVID-19 infection), shortness of breath (today and before COVID-19 infection), fatigue, functional limitations (today and before COVID-19 infection), anxiety and depression, and loneliness. In addition to socio-demographic questions, the questionnaire includes questions regarding potential risk or protective factors: COVID-19 vaccination status, health history (e.g. underlying chronic diseases), and health behaviours.

The follow-up questionnaires aimed to assess, every 3 months, the evolution in the health status and factors potentially associated. To follow the evolution, it included several topics collected in baseline: health-related quality of life, shortness of breath, fatigue, functional limitations, anxiety and depression, and loneliness. These questionnaires also aimed to collect information on the long-term effects of a COVID-19 infection via the following topics: COVID-19 persistent or new symptoms (i.e. list of potential PCC symptoms, medical complications (such as pulmonary embolism or deep vein thrombosis), change in employment and income status. Finally, the follow-up questionnaires also included questions regarding COVID-19 vaccination status, health care use (e.g. hospitalisations, consultations) and lifestyle changes.

### Matched-control group

In order to compare the physical, mental and social health outcomes of the cohort to a sample of people not infected with COVID-19, a matched sub-sample was selected from the Belgian Health Interview Survey (BHIS). Indeed, this study used several standardized tools identical to those used in the BHIS. The outcomes we have in both this study and the matched-control group are shown in Table 1. The BHIS is the leading health survey in Belgium [33], carried out every 4–5 years since 1997 by Sciensano, the Belgian Institute of Public Health, and aligned with the European Health Interview Survey. The last survey was in 2018 and the next one is scheduled for 2023. Using a representative sample, the BHIS collects information on the health and well-being, health behaviour and lifestyle, health care use, and physical and social environment of the population and living in Belgium. Based on the distribution by age, sex, and level of education of the current cohort of this study, a sub-sample with a similar sociodemographic distribution was extracted from the 2018 BHIS (*n* = 3.263 out of 11.611) using a stratified random sampling method. This matched-control group will be adapted according to the evolution of the profile of the cohort. In addition, the matched-control group will be updated with data from the next BHIS in 2023.

### Data management

Study data obtained via de online survey on LimeSurvey are transferred to a secured SAS database hosted at Sciensano with restricted access to the researchers involved in the study. Personal data were stored in a separate secure file accessible only to project coordinators. Data management and analysis were performed in SAS® 9.4.

### Characteristics of participants

Table 2 presents the characteristics of participants in the different stages of the study, distinguishing the recruitment in the cohort from the follow-up of the cohort. Data on the eligible population and those who agreed to receive the SMS were retrieved from the national contact tracing database.

**Table 2:** Characteristics of participants in the different stages of the study

	**Recruitment in the cohort Time: acute phase of the infection (from 29/04/21 to 01/11/21)**				**Follow-up of the cohort Time: 3 months after infection (from 29/07/21 to 01/11/21**		
	Eligible participants	Participants who agreed to receive the SMS	Participants who completed the baseline questionnaire	Participants who agreed to be followed-up	Participants contacted for the first follow-up questionnaire	Participants who completed the first follow-up questionnaire	Participants in the first follow-up who agreed to participate in the second follow-up
	National contact tracing data		Study data		Study data		
	*n* = 225,119	*n* = 66,645	*n* = 10,708	*n* = 8,438	*n* = 3,240 (remaining 5,198 participants will be contacted after 01/11/21)	*n* = 2,101	*n* = 1,659
Age groups, n (%)							
•18–25	42,048 (18.7)	14,132 (21.2)	1,660 (15.5)	1,047 (12.4)	518 (16.0)	282 (13.4)	189 (11.4)
•26–45	102,346 (45.5)	31,894 (47.9)	5,130 (47.9)	4,076 (48.3)	1,527 (47.1)	1,010 (48.0)	785 (47.3)
•46–65	60,742 (27.0)	17,860 (26.8)	3,534 (33.0)	2,978 (35.3)	1,111 (34.3)	760 (36.3)	643 (38.7)
•66 85	17,549 (7.8)	2,672 (4.0)	374 (3.5)	329 (3.9)	81 (2.5)	47 (2.2)	42 (2.5)
•86 +	2,434 (1.0)	87 (0.1)	10 (0.1)	8 (0.1)	3 (0.1)	2 (0.1)	0 (0.0)
Sex, women, n (%)	120,031 (53.3)	35,624 (53.4)	6,489 (60.6)	5,213 (61.8)	1,950 (60.2)	1,315 (62.6)	1,075 (64.8)
At least one acute COVID-19 symptom, n (%)	178,040 (79.1)	56,046 (84.1)	9,894 (92.4)	7,754 (91.9)			
Persistent symptoms, n (%)							
•0						1,093 (52.0)	576 (34.7)
•1						274 (13.0)	328 (19.7)
•2						233 (11.1)	302 (18.2)
•3						132 (6.3)	152 (9.2)
•4 or more						369 (17.6)	301 (18.2)

Through the different stages of the recruitment in the cohort during the acute phase of infection, we observed a decrease in the proportion of participants between 18–25 years (from 19% among eligible participants to 12% among participants who agreed to be followed-up) and 66–85 years (from 8 to 4%). The proportion of women increased from 53% among eligible participants to 62% among participants who agreed to be followed-up. Regarding the acute COVID-19 symptoms, the proportion of participants reporting at least one acute COVID-19 symptom increased from 79 to 92%.

The evolution of participant characteristics through the cohort 3-month follow-up stages was similar. Between the profile of the participants contacted for the first follow-up questionnaire and that of the participants in the first follow-up who agreed to participate in the second follow-up, we observed: (1) a decrease in the proportion of participants between 18–25 years (from 16 to 11%), (2) an increase in the proportion of women (from 60 to 65%), and (3) an increase in the proportion of participants reporting persistent COVID-19 symptoms 3 months after infection. Regarding persistent COVID-19 symptoms between participants who completed the first 3-month follow-up questionnaire and those who agreed to participate in the second 6-month follow-up, the proportion reporting no persistent symptom decreased (from 52 to 35%) and the proportion reporting 1 or 2 persistent symptoms increased (respectively from 13 to 20% and from 11 to 18%). The proportion reporting 3 or 4 or more persistent symptoms remained relatively stable (respectively from 6 to 9% and from 18 to 18%).

Additional analyses were performed on the profile of participants who dropped out of the 3-month cohort and those who completed the 3-month follow-up questionnaire and these are shown in Supplementary Table 1. The results confirm the data presented in Table 2: there was a significant difference in the distribution by age groups (*p* < 0.001) and sex (*p* < 0.001), with a higher proportion of people aged 18 to 25 (21% vs. 13%) and a lower proportion of women (56% vs. 63%) among the participants who dropped out than among those who completed the first follow-up questionnaire. Conversely, additional analyses showed that there was no significant difference between the two groups in terms of level of education (*p* = 0.45), presence of a chronic disease (*p* = 0.31), and COVID-19 vaccination status (*p* = 0.19).

Further additional analysis shown in Supplementary Table 2, presents a comparison of the sociodemographic characteristics of (1) the general population in Belgium aged 18 and over, (2) the eligible population (i.e. all adults infected with COVID-19 between 29/04/21 ad 01/11/21), and (3) the participants who completed the first 3-month follow-up questionnaire. Compared to the general adult population, there was a higher proportion among the eligible population of people aged 18 to 25 (11% vs. 19%) and 26 to 45 (32% vs. 45%). The proportion of women in the two groups was relatively similar (51% vs. 53%). The proportion of people aged 26 to 45 (48%) and women (63%) increased among the participants who completed the first follow-up questionnaire 3 months after infection.

### Assessment and description of post COVID-19 condition

Several studies have shown that people with Post COVID-19 Condition (PCC) tend to report many and heterogeneous symptoms [8, 9]. To assess PCC, it is therefore important to have an exhaustive list of possible symptoms. Indeed, one systematic review found that the prevalence of PCC ranged from 4.7% to 80% between studies depending on the definition used and symptoms included [7]. In this study, a list of 28 potential PCC symptoms (see Table 3) is presented to participants based on recently published guidelines [6, 11, 34, 35]. According to the evolution of the knowledge of PCC, some symptoms or complications may be added to the follow-up questionnaire. Participants also had the option of indicating that they suffered from another symptom not included in the list. Currently, 2.8% of the participants (see Table 3) have selected this “other symptom” category, indicating that the list of potential symptoms is relatively comprehensive.

**Table 3:** Description of COVID-19 variables three months after infection (*n* = 2,101)

Participants who completed the 3-month follow-up questionnaire (November 1, 2021, *n* = 2,101)			
Symptoms after 3 months, n (%) weighted^**a**^ *%*			
Fatigue/exhaustion	498 (23.7) *20.8*	Tingling feeling	84 (4.0) *3.3*
Headache	264 (12.6) *10.9*	Chest pain	84 (4.0) *3.4*
Memory problems	258 (12.3) *10.3*	Ringing in ears	77 (3.7) *3.0*
Muscle pain	229 (10.9) *6.3*	Loss of appetite	67 (3.2) *2.6*
Shortness of breath	220 (10.5) *8.8*	Stomach pain	65 (3.1) *2.8*
Sleeping problems	205 (9.8) *8.2*	Skin rashes	63 (3.0) *2.7*
Loss of smell	184 (8.8) *7.8*	Others	58 (2.8) *2.5*
Joint pain	153 (7.3) *6.8*	General malaise	54 (2.6) *2.1*
Loss of taste	128 (6.1) *4.9*	Confusion	52 (2.5) *1.8*
Dizziness	121 (5.8) *4.6*	Weight loss	48 (2.3) *2.2*
Palpitations	111 (5.3) *4.4*	Problems speaking	46 (2.2) *1.6*
Constipation	102 (4.9) *3.9*	Problems swallowing	10 (0.5) *0.2*
Persistent cough	99 (4.7) *4.8*	Swelling/oedema	10 (0.5) *0.1*
Problems seeing	95 (4.5) *3.5*	Incontinence	6 (0.3) *0.1*
Average number of symptoms 3 months after infection, mean (SD) weighted^**a**^ *mean*			3.4 (2.8) *2.4*
Case definition of PCC (i.e. at least one symptom related to the COVID-19 infection three months after it), n (%) weighted^**a**^ *%*			
•Yes			1,008 (48.0) *42.5*
•No			1,093 (52.0) *57.5*
Self-perceived recovery from COVID-19 after 3 months, n (%) weighted^**a**^ *%*			
•Completely recovered			1,180 (56.2) *63.8*
•Somewhat yes			531 (25.3) *22.1*
•Neither yes nor no			194 (9.2) *7.0*
•Not really recovered			131 (6.2) *4.8*
•Not feel recovered at all			65 (3.1) *2.3*
Diagnosed with PCC by a healthcare professional, n (%) weighted^**a**^ *%*			
•Yes			551 (26.2) *19.8*
•No			1,550 (73.8) *80.2*

^a^ Post-stratification weighting based on the distribution of the eligible population by age, sex, and proportion having at least one acute symptom of COVID-19

Table 3 presents a description of the main variables related to COVID-19 three months after infection.

The results of the post-stratification weighting based on the distribution of the eligible population by age, sex, and proportion having at least one acute symptom of COVID-19 are also presented in Table 3. Three months after COVID-19 infection, participants had an average of 3 different symptoms. The most common symptoms at 3 months were fatigue/exhaustion (crude 24%, weighted 21%), headache (crude 13%, weighted 11%), memory problems (crude 12%, weighted 10%), muscle pain (crude 11%, weighted 6%), shortness of breath (crude 10%, weighted 9%), and sleeping problems (crude 10%, weighted 8%). The definition we used to identify people who suffer from PCC 3 months after their COVID-19 infection is that of the National Institute for Health and Care Excellence (NICE): “Signs and symptoms developed during or after an infection consistent with COVID-19, continue for more than 12 weeks and are not explained by an alternative diagnosis” [11]. Based on this definition, 48% (*n* = 1,008) of participants in the cohort appeared to fit the proposed case definition of PCC (weighted 42%). Two other variables are available and could be used in the future to refine the definition: whether people feel recovered from COVID-19 and whether they have been diagnosed with PCC by a healthcare professional. Among the participants in the 3-month cohort (*n* = 2,101), 3% (weighted 2%) did “not feel recovered at all” from COVID-19, 6% (weighted 5%) “not really recovered, 9% (weighted 7%) “neither yes nor no,” 25% (weighted 22%) “somewhat yes”, and 56% (weighted 64%) “completely recovered”. Finally, 26% were diagnosed with PCC by a healthcare professional (weighted 20%).

## Discussion

The main objective of this online longitudinal cohort study is to study the long term evolution of the physical, mental and social health of adults who have been tested positive with COVID-19 in Belgium, and assess the factors associated with a(n) (un)favourable evolution.

In terms of recruitment of participants, while 30% of the eligible population (i.e. all adults infected with COVID-19 between 29/04/21 and 01/11/21) agreed to receive more information about the study and the link to the questionnaire via SMS, only 16% of those who received the SMS completed the baseline online questionnaire. This accounts to a participation rate in the first baseline questionnaire of 5% of the eligible population. Analyses on the profile of participants in the different stages of the recruitment in the cohort (at the time of COVID-19 infection) revealed a higher proportion of people between 46–65 years, of women, and of people reporting at least one acute COVID-19 symptom among participants who agreed to be followed-up in comparison to eligible participants. Conversely, the proportion of people between 18–25 years and 66–85 years decreased as the recruitment phases in the cohort progressed. The literature on surveys has already shown that extreme age groups and men are less likely to participate in online surveys, resulting in sample selection bias [36, 37]. Besides, our results showed that people who reported at least one acute COVID-19 symptom were more likely to complete the baseline questionnaire, also inducing a selection bias in the initial cohort. Therefore, post-stratification weights were used and will be used for future analysis to adjust for the distribution of the eligible population. The variables available for the eligible population (data from national tracing centers) that were included for the calculation of post-stratification weights were age, sex, and having at least one acute symptom of COVID-19. The weighting confirmed an overestimation of the proportion of PCC and its symptoms in the cohort (e.g. proportion of fatigue/exhaustion from 24 to 21%).

Regarding the follow-up of the cohort, among the participants contacted by email 3 months after their COVID-19 infection, 65% completed the follow-up questionnaire. Finally, 21% of them indicated that they no longer wanted to be followed over time. The profile of participants also changed as the follow-up progressed. Indeed, among participants contacted to complete the first follow-up questionnaire 3 months after their infection, women and people reporting persistent COVID-19 symptoms were more likely to complete the questionnaire and agree to be contacted for the next follow-up. This last result is important because it implies an overestimation in the proportion of Post-COVID-19 Condition (PCC) in the sample of the cohort, and it is possible that this phenomenon increases after each 3-month follow-up. Conversely, additional analyses presented in Supplementary Table 1 showed that there was no significant difference between participants who dropped out of the 3-month cohort and those who completed the 3-month follow-up questionnaire in terms of level of education, presence of chronic disease, and COVID-19 vaccination status. These results tend to show that the selection bias in the follow-up of the cohort was essentially linked to age and sex.

There is currently no consensus on the definition of PCC and people who suffer from it, tend to report numerous and heterogeneous symptoms. In this study, we are working with the most comprehensive possible list of potential PCC symptoms and the following definition: “Signs and symptoms developed during or after an infection consistent with COVID-19, continue for more than 12 weeks and not explained by an alternative diagnosis” [11]. Based on the list of symptoms and this definition, 48% of participants in the cohort appeared to fit the proposed case definition of PCC (weighted 42.5%). The symptoms are self-reported by participants so we cannot ensure that they are not explained by an alternative diagnosis. This proportion is close to that reported by a recent meta-analysis carried out on 33 studies on hospitalised and non-hospitalised COVID-19 survivors and showed that 46% of the people had at least one PCC symptom three months after acute infection [8]. Finally, in October 2021, WHO developed the following clinical case definition of PCC by Delphi methodology with patients, researchers and others: *“Post COVID-19 condition occurs in individuals with a history of probable or confirmed SARS CoV-2 infection, usually 3 months from the onset of COVID-19 with symptoms and that last for at least 2 months and cannot be explained by an alternative diagnosis. Common symptoms include fatigue, shortness of breath, cognitive dysfunction but also others and generally have an impact on everyday functioning. Symptoms may be new onset following initial recovery from an acute COVID-19 episode or persist from the initial illness. Symptoms may also fluctuate or relapse over time.”* [6]. This definition, in addition to being more precise, adds a dimension of “persistence of symptoms”, these must have persisted for at least two months. As part of the COVIMPACT study, follow-up data 6 months after infection will allow us to assess the persistence of symptoms beyond 3 months.

### Comparison with other studies and strengths and limitations

In light of other studies on PCC, the COVIMPACT study has some strengths and limitations. One meta-analysis [8] included 22 studies on PCC in hospitalised people and 12 in non-hospitalised people allows our study design to be compared to others. The average follow-up period for participants in the studies was between 30 and 90 days after infection or hospitalisation. Among the studies in non-hospitalised people, 5 recontacted participants by telephone, 2 via face-to-face interaction, 1 by post, and 4 by electronic means or website. By telephone, most studies had a small sample size, the largest being 510 people recontacted 40 days after infection (median), with 24% lost to follow-up [38]. The two face-to-face studies in the USA [39] and Mexico [40] had respectively a convenience sample of 96 and 219 participants recontacted 115 and 30 days after infection (median). Regarding the study using post, 938 participants were invited 117 days after infection (median) and 451 answered the questionnaire (response rate of 48%) [20]. The two largest sample sizes were found in two of the four studies that collected data electronically or via a website. The first was a multi-country study with data on 4,182 participants followed up 30 or 60 days after infection via the “COVID Symptom Study” mobile app [41]. The second study, in the Netherlands, had a sample of 2,113 participants followed up 80 days (median) after infection and recruited through Facebook groups for people with persistent COVID-19 symptoms and from a panel of people who registered on a website of the Lung Foundation Netherland [14]. The design of data collection in these two studies may lead to significant selection bias in the sample. For example, participants in both studies were disproportionately female (respectively 72% and 85%).

The COVIMPACT study has three main strengths. First, the majority of studies on PCC have followed up people after hospitalisation, however, PCC also affects people with moderate symptoms or who were asymptomatic during the acute phase of the infection [14, 20]. In the COVIMPACT study, the entry point is to have been tested positive with COVID-19 and the eligible population was all adult living in Belgium with a COVID-19 infection confirmed via a laboratory test during the study period. The second strength of this study is the follow-up of participants every 3 months and up to two years. Most studies on PCC had a follow-up period between 30 and 90 days after infection or hospitalisation [8] and a meta-analyses [22] of 18 studies with a one-year follow-up found that they had a limited sample size, the smallest being 83 people and the largest being 543 people. Therefore, the COVIMPACT study will provide timely information to stakeholders such as policymakers, health practitioners and long haulers, on the short, medium and long term impacts of a COVID-19 infection. Finally, the third strength of this study is its global approach to the consequences of a COVID-19 infection on the health of individuals, with measures on different dimensions of their physical, mental and social health. This information allows us to identify people at risk in these different dimensions of health as well as the factors associated with a(n) (un)favourable evolution of these different dimensions. In addition, our matched-control group will allow us to compare the physical, mental and social health outcomes of the cohort to a sample of people not infected with COVID-19. Indeed, a systematic review of studies on PCC found that there was a lack of case–control studies or studies with a matched non-COVID-19 group (1).

This study also has several potential limitations. As previously explained, it allows us to study the evolution of the physical, mental and social health of people infected with COVID-19, and to compare these outcomes with a matched-control group of the 2018 general Belgian population not infected with COVID-19. However, this 2018 control group was not exposed to the global health crisis and to the measures taken to limit the spread of the virus (e.g. lockdown, restriction of social contacts, etc.). Crisis and measures that independently of a COVID-19 infection can have effects on the physical, mental and social health of the general population. Therefore, the matched-control group will be updated with data from the next Belgian Health Interview Survey in 2023. In addition, PCC symptoms are common to many other diseases and infections that affect the general population and we do not have information on the frequency of these symptoms in the general population not infected with COVID-19. Although participants self-reported that these symptoms were related to their COVID-19 infection, we cannot perform sensitivity analyses with a control group. Second, a selection bias occurs at the recruitment phase as some people do not have mobile phones to receive the SMS, so they will have no access to the link. In addition, in 2020, still 10% of the population in Belgium had no access to an internet connection and some groups may not have the skills to complete online surveys. Finally, this study is also exposed to differential loss to follow-up because our results showed that people who have long lasting symptoms are more likely to stay in the cohort and the loss to follow-up may more likely be participants who have no long lasting symptoms, or on the contrary people who have died.

## Supplementary Information

**Additional file 1:** **Supplementary Table 1.** Sociodemographiccharacteristics of (1) participantswho dropped out from the 3-month cohort and (2) participantswho completed the 3-month follow-up questionnaire.

**Additional file 2:** **Supplementary Table 2.** Sociodemographiccharacteristics of (1) the general population in Belgium, (2) eligibleparticipants, and (3) participants who completed the first 3-month follow-upquestionnaire.

## Data Availability

The data of this study are available from the corresponding author upon reasonable request.

## Abbreviations

PCC: Post COVID-19 Condition
SARS-CoV-2: Severe Acute Respiratory Syndrome Coronavirus 2
WHO: World Health Organization
GAD-7: General Anxiety Disorder-7
PHQ-9: Patient Health Questionnaire-9
NICE: National Institute for health and Care Excellence
