# Supplementary material for: Post COVID-19 condition and its physical, mental and social implications: protocol of a 2-year longitudinal cohort study in the Belgian adult population
Source: Arch Public Health. 2022 Jun 4;80:151. doi: 10.1186/s13690-022-00906-2 (PMC9166244; doi:10.1186/s13690-022-00906-2)
Supplement: Supplementary file 1 — Additional file 1: Supplementary Table 1. Sociodemographiccharacteristics of (1) participantswho dropped out from the 3-month cohort and (2) participantswho completed the 3-month follow-up questionnaire. [file 13690_2022_906_MOESM1_ESM.docx]

Supplementary Table 1: Sociodemographic characteristics of (1) participants who dropped out from the 3-month cohort and (2) participants who completed the 3-month follow-up questionnaire

| Follow-up of the cohort  Time: 3 months after infection (from 29/07/21 to 01/11/21) | | |  |
| --- | --- | --- | --- |
|  | Participants who dropped out from the 3-month cohort | Participants who completed the 3-month follow-up questionnaire | χ2 (p-value) |
|  | n = 1,139 | n = 2,101 |  |
| Age groups, n (%)   - 18-25 - 26-45 - 46-65 - 66-85 - 86+ | 236 (20.7)  517 (45.4)  351 (30.8)  34 (3.0)  1 (0.1) | 282 (13.4)  1,010 (48.0)  760 (36.3)  47 (2.2)  2 (0.1) | 33.6 (<0.001) |
| Sex, women, n (%) | 635 (55.7) | 1,315 (62.6) | 14.4 (<0.001) |
| Educational status, n (%)   - Secondary school or below - Higher education | 318 (27.9)  821 (72.1) | 592 (28.2)  1509 (71.8) | 1.02 (0.45) |
| Chronic disease, yes, n (%) | 84 (7.4) | 147 (7.0) | 1.15 (0.31) |
| COVID-19 vaccination status at the time of infection   - None - Partial - Complete | 376 (33.0)  124 (10.9)  639 (56.1) | 679 (32.3)  241 (11.5)  1181 (56.2) | 2.33 (0.19) |
