# Supplementary material for: Post COVID-19 condition and its physical, mental and social implications: protocol of a 2-year longitudinal cohort study in the Belgian adult population
Source: Arch Public Health. 2022 Jun 4;80:151. doi: 10.1186/s13690-022-00906-2 (PMC9166244; doi:10.1186/s13690-022-00906-2)
Supplement: Supplementary file 2 — Additional file 2: Supplementary Table 2. Sociodemographiccharacteristics of (1) the general population in Belgium, (2) eligibleparticipants, and (3) participants who completed the first 3-month follow-upquestionnaire. [file 13690_2022_906_MOESM2_ESM.docx]

Supplementary Table 2: Sociodemographic characteristics of (1) the general population in Belgium, (2) eligible participants, and (3) participants who completed the first 3-month follow-up questionnaire

|  | Population Belgium 2021, aged 18+ | Eligible participants  (aged 18+, living in Belgium, with a COVID-19 infection between 29/04/21 ad 01/11/21) | Participants who completed the first 3-month follow-up questionnaire |
| --- | --- | --- | --- |
|  | n = 9,209,116 | n = 225,119 | n = 2,101 |
| Age groups, n (%)   - 18-25 - 26-45 - 46-65 - 66-85 - 86+ | 1,061,765 (11.5)  2,984,524 (32.4)  3,068,754 (33.3)  1,812,679 (19.7)  281,394 (3.1) | 42,048 (18.7)  102,346 (45.5)  60,742 (27.0)  17,549 (7.8)  2,434 (1.0) | 282 (13.4)  1,010 (48.0)  760 (36.3)  47 (2.2)  2 (0.1) |
| Sex, women,  n (%) | 9,209,116 (51.2) | 120,031 (53.3) | 1,315 (62.6) |
